# Accommodative Response to Asymmetrical Accommodative Stimuli

**DOI:** 10.3390/vision9010022

**Published:** 2025-03-12

**Authors:** Vasili Marshev, Jean-Louis de Bougrenet de la Tocnaye, Vincent Nourrit

**Affiliations:** 1 Optics Department, IMT Atlantique, 29238 Brest CEDEX 03, France; vasilimarshev@gmail.com (V.M.); jl.debougrenet@imt-atlantique.fr (J.-L.d.B.d.l.T.); 2 LaTIM (Laboratory of Medical Information Processing), Brest Institute of Biological and Health Research, 29200 Brest, France

**Keywords:** accommodation response, aniso-accommodation, haploscope, spatial frequency

## Abstract

Recent advancements in head-up-displays have increased the number of instances where the visual system may face a different accommodative demand for each eye. A limited number of studies on aniso-accommodation exist, reporting contradictory results. We tested the natural capacity of observers to aniso-accommodate anisometropic stimuli. A dichoptic task allowed us to account for certain confounds, including high-level accommodation control. A 2AFC visual task was used, where participants judged if two overlapping sinusoidal gratings, presented dichoptically, had the same orientation. The gratings’ spatial frequency could be 1, 4 or 10 c/deg. The accommodative demand for each eye could be independently set to 2D or 4D. The accommodative response for each eye was recorded using an autorefractometer. Higher spatial frequencies and anisometropy had a negative impact on task accuracy. Contrary to expectations, spatial frequencies had no significant impact on accommodation response. The accommodation response to anisometropic stimuli was equal in the two eyes and leaned toward the lower of two demands. Our results confirm that when presented with asymmetrical accommodation demand, the two eyes tend to keep the same refractive power even in a dichoptic-requiring task. They also contradict the guidance of accommodation by spatial frequency in sinusoidal gratings. The visual task provided an objective measure of subjects’ performance, allowing for these conclusions to be drawn.

## 1. Introduction

In recent years, technological advancements have led to the development of various virtual reality and augmented reality designs. These devices may cause the visual system to function unnaturally, thus raising a number of questions in terms of comfort, health and safety (e.g., accommodation–vergence conflict [1,2,3], vertical disparity [4], restricted field of view [5], cybersickness [6,7], etc.). Among these potential issues, unequal accommodation demand (AD) has received little attention, although it may appear in modern displays as a design feature [8], such as in monocular augmented reality headsets, or in virtual reality due to incorrect alignment between the eye and the high-power lenses [9].

In the literature on aniso-accommodation, several studies have produced conflicting results. Marran and Schor [10] reported an increasing difference between the left and right eye accommodation response (AR) proportional to the difference in AD. Conversely, in Koh and Charman’s study [11], when presented with different AD for each eye, subjects’ AR was kept the same in the two eyes closer to the lowest of the two ADs. Flitcroft et al. [12] measured an AR to anisometropic stimuli that was close to a compromise between the two ADs. Vincent et al.’s results [13] suggested a systematically increased AR in the dominant eye when viewing anisometropic stimuli.

An inspection of the methods of the studies on aniso-accommodation shows an important detail in Marran and Schor’s report due to which it differed from other studies. Before the main measurement, subjects went through an unlimited training period during which they trained to exhibit aniso-AR. Training is known to not just influence AR, but also help elicit it in darkness [14]. Similarly, we know that voluntary control can alter AR, reducing the influence of the features of the stimulus, such as spatial frequency (SF) [15].

The aim of this study was therefore to answer the following question: what will be the AR to anisometropic stimuli, and how will it be influenced by SF, if we ensure that subjects exert sufficient accommodative effort to perform the task? Our hypothesis was that the difference in the results on aniso-accommodation reported in the literature could be due to the influence of higher-level control factors on accommodation, which had not been properly controlled previously, and that by controlling for this effect, the impact of each parameter (AD, SF) on AR will be significant. To test our hypothesis, we therefore measured the aniso-accommodation capacity (in a larger number of subjects, most studies had seven or fewer subjects) and studied how it can be influenced by AD and SF while controlling for higher-level factors. To do so, we measured subjects’ performance in a visual task while at the same time recording their AR. Our assumption was that by filtering out blocks of trials with low accuracy in the task (<75%), we would ensure that subjects exerted sufficient accommodative effort to perform the task.

## 2. Materials and Methods

The task consisted of fusing two high-contrast gratings presented dichoptically and judging whether they had the same orientation or not. Two difficulty levels were implemented. For the easy difficulty, the orientation difference between the two gratings could be 0° or 30°. For the difficult level, this difference could be 0° or 15°. Gratings could be presented at the same distance (isometropic) or with a 2D difference (anisometropic).

We chose sinusoidal gratings because they allowed us to freely vary the level of detail (through SF) and orientation (e.g., square gratings would have produced graphical artifacts when presented at oblique orientations). The 2D accommodation difference was chosen in agreement with the literature (the upper limit in previous studies was 2D or 3D [10,11,12,13]) and to potentially stimulate a significant difference in AR.

The following subsections present the stereoscopic display, stimuli and procedure in more detail.

### 2.1. Haploscope Setup

**Figure 1:**
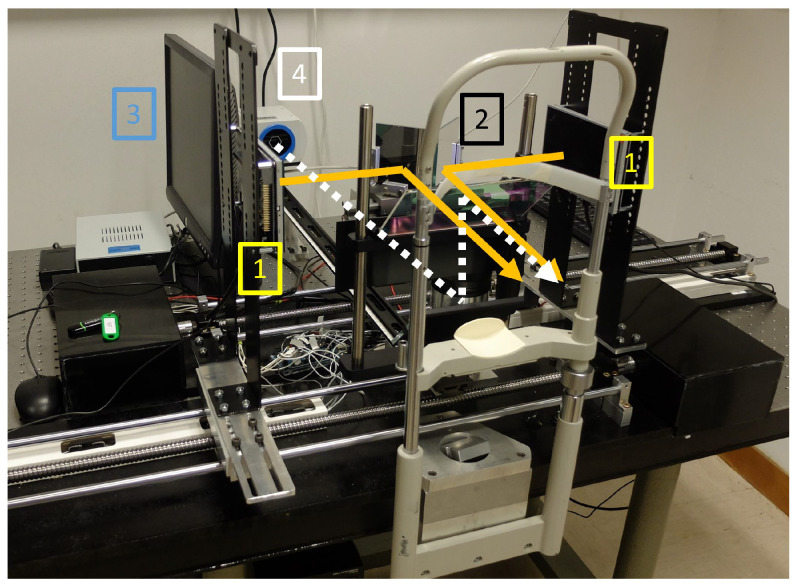
The haploscope used in the study. A pair of mirrors (2) at 45 degree angles to the user’s eyes allow us to present a different display (1) to each eye. The screens are mounted on motorized rails (linear screw and two guides for each side): item (3) (the large screen monitor on the left is the one used to control the experiment). The subject’s accommodation is recorded at the same time with a refractometer (4). The solid line illustrates the optical path from the stimuli to the subject’s eyes, while the dashed line illustrates the optical path for the accommodation measurement.

In order to measure aniso-accommodative stimuli in a controlled environment, we constructed a stereoscope system with motorized screen supports coupled to an autorefractometer. The instrument is depicted in Figure 1. The participant positioned their face on the chin rest and looked at the screens (see 1 in Figure 1) where the stimuli could be seen through a pair of first-surface mirrors (see 2 in Figure 1) rotated at 45° to the frontal axis around the longitudinal axis.

Each screen (MCT070HDMI, Midas, 1024 × 600 pixels, 154 × 86 mm) was attached to a support which could move on a set of rails (see 3 on Figure 1). The two rails were positioned in front of the observer parallel to each other and perpendicular to the line of sight. Two viewing distances were used: 2D and 4D. We chose to change the AD by changing the screen position rather than using a Badal system with positive and negative lenses to avoid the interference caused by the lens type [16] and possible optical aberrations in the measurements introduced by the lenses.

At any viewing distance, stimuli appeared in the medial plane of the subject. Thanks to this, the accommodation and convergence demands of the stimuli were always in agreement. This was achieved through the following configuration. The mirrors were placed as close to each other as possible. The edge where the mirrors’ surface planes crossed was aligned with the medial plane of the observer. The same edge was also aligned with the axis between the screens’ centres in the plane parallel to the frontal plane of the observer.

A PowerRef3 autorefractometer (Plusoptix) (see 4 on Figure 1) was positioned in front of the subject with the help of a beam splitter. This is an infrared refractometer [17] that may be used to obtain objective measures of accommodation both monocularly and binocularly [18].

### 2.2. Stimuli and Procedure

**Figure 2:**
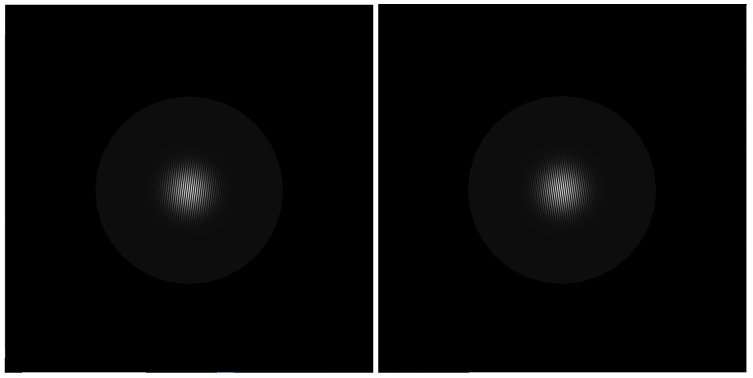
Stimuli shown in the experiment. Circular sinusoidal gratings were shown monocularly. The (**left**) and (**right**) images (shown side by side here) were fused using the gray ring around the gratings to help subjects align them to compare the gratings’ orientations.

In each trial, two monocular circular sinusoidal gratings were presented (94% contrast, maximum 200.8 cd/m^2^, minimum 6.4 cd/m^2^; see Figure 2), 2 deg. of v.a. in diameter. They appeared in the center of the screen (one for each eye, as depicted in Figure 1). Around each grating, a dark-gray (17.7 cd/m^2^) ring was drawn, 9 deg. of v.a. in diameter. The ring served as a fusing stimulus. Around the fusing ring was a black background (6.4 cd/m^2^).

Each of the gratings were slightly tilted away from the vertical (5°–25°) and tilted away from each other so that the angle between the two gratings was always 0°, 15° or 30°. To achieve this, the orientation of a randomly chosen grating (left or right) was assigned a random rotation between 5° and 10° or 5° and 25°, depending on if the difference between the two gratings to be achieved was 15° or 30°. The second grating orientation was shifted accordingly. As a result, the two gratings’ orientations, when different, were always on the opposite sides of the vertical.

The gratings were shown until the participant indicated whether the two gratings’ orientations were the same or not by pressing one of two buttons on a joystick. Then, the stimuli were replaced with a white-noise mask for one second until the next trial began.

The trials were grouped into blocks of 16 trials. In each block, in half of trials, the two gratings’ orientations were equal. In the other half of trials, the gratings had different orientations. Blocks in which half of trials had gratings with equal orientations (0° difference) and the other half had gratings with a 30° difference were called “easy”. Blocks in which half of the trials had a 0° difference and the other half had a 15° difference were called “hard”.

All gratings in a group of 16 consecutive trials (block) also had the same SF (1, 4, 10 c/deg, see Appendix B). The screen distance was also kept the same throughout a block, i.e., either 50 cm from both the left and right targets (isometropic 2D AD condition), 25 cm from both targets (isometropic 4D AD condition), the left target at 25 cm and the right target at 50 cm (anisometropic OS > OD condition), and the left target at 50 cm and the right target at 25 cm (anisometropic OS < OD condition). Whatever the AD, the size and SF of the gratings were rescaled to be the same across all conditions from the viewer’s point of view.

In the instruction, it was emphasized to the subjects that they were required to look through the two mirrors, with each eye looking at its dedicated screen. It was pointed out that they must not look at the targets sequentially by closing one eye at a time or view both targets through one mirror.

After receiving the instructions, subjects underwent training which included at least two blocks of trials. Before the experiment began, subjects adapted to the dark for five minutes. Frequent breaks were offered to alleviate any eye fatigue.

Vertical displacement due to individual differences was corrected using the following procedure. With their chin on the chin rest, the subject viewed the two screens in an anisometropic condition (the left screen in 4D and the right screen in 2D). No gratings were shown, but on top of the fusing ring on each screen, a red dot was presented. Participants adjusted the height of the chin rest until the left and right red dots were aligned.

### 2.3. Analyses

The AR values obtained with the PowerRef were smoothed using 10-point running averaging (taking the average of the last 10 successful measurements (i.e., over a 0.2 s interval)) to reduce noise. A repeated measures ANOVA was conducted in R studio [19] with all predictors as within-subject factors.

To assess the impact of SF on AR, only isometropic blocks, i.e., when AD was the same for each eye, were considered. The average AR of the two eyes across each block was then used as the dependent variable, and SF and AD were predictors (3 × 2).

To assess whether or not subjects could aniso-accommodate, the difference between the AR of the left and right eyes was used as the dependent variable, and AD difference (−2; 0; 2) as the independent variable. To obtain each participant’s natural anisometropia, the difference between the left and right eye AR in the isometropic conditions was averaged. It was then subtracted from all AR difference measurements of a given subject.

Two assumptions were made. First, participants’ effort may be controlled by the presence of the visual task itself. This would predict the highest AR in the high-SF condition. Also, filtering out blocks with low task accuracy (<75%) may show a clearer relation between SF and AR. Second, the task can facilitate subjects to elicit aniso-AR and filtering out blocks with low accuracy (<75%) would help clarifying participants’ capability to aniso-accommodate.

### 2.4. Subjects

We performed a power analysis to identify the necessary sample size. To test the impact of SF on AR (3 × 2), we employed PANGEA software [20]. Using data from [21] (p. 9) reporting the AR to SFs of 0.5 and 4 c/deg, we calculated Cohen’s *d* effect size to be 0.729. Given two replicate values per condition (two angles between the gratings), 13 participants were required to achieve a power of 0.95.

The sample size required for testing the aniso-accommodation hypothesis was assessed through data simulation based on a pilot study and a previously reported experiment [22]. A detailed description of the procedure can be found in Appendix A. According to this power analysis, 14 participants were required to reach a power of 0.95.

Only participants with no binocular vision issues or ocular pathologies were included in the study. Subjects with prescribed glasses or contact lenses wore their normal corrective lenses.

Out of twenty recruited subjects (age *M* = 25; *SD* = 4; 7 females), data of five subjects (all male) were discarded due to poor pupil recognition by the autorefractometer, resulting in large number of missing AR readings.

This study was carried out in accordance with the tenets of the Declaration of Helsinki. Written informed consent was obtained from all participants. The study was carried out between 2 July and 25 September 2020.

## 3. Results

### 3.1. Task Accuracy

**Figure 3:**
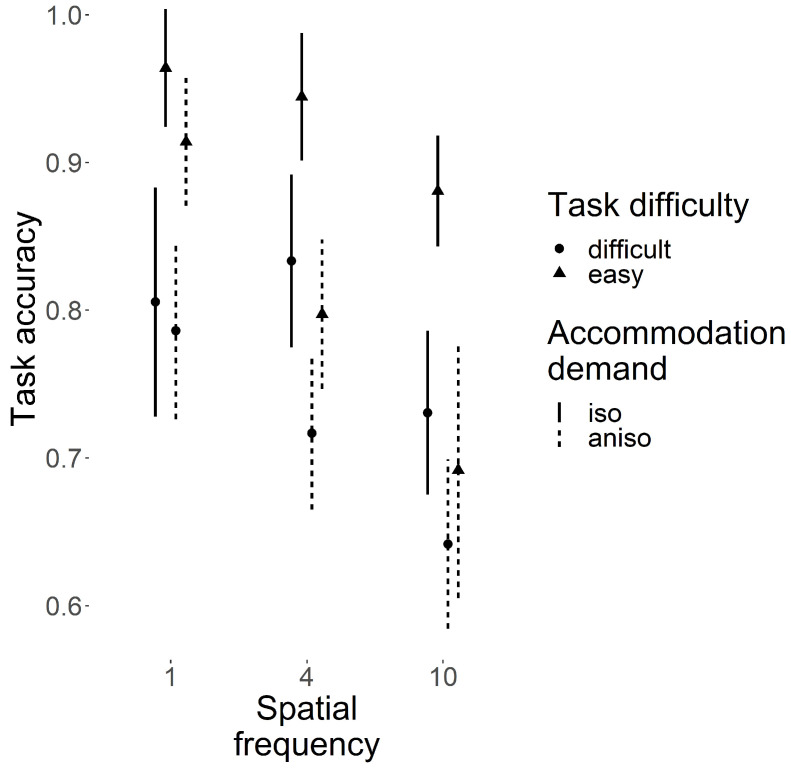
The effect of stimulus parameters on task accuracy. The ordinate axis represents the average correct response rate per block, and the abscissa axis represents the stimulus spatial frequency. Each data point (plain disks or triangles, denoting whether the task is easy or difficult, respectively) represents the average accuracy across all participants. Error bars represent 95% confidence intervals (solid lines for the isometropic conditions, dashed lines for the anisometropic conditions). The main effects of accommodation demand symmetry, spatial frequency and task difficulty, as well as the interaction of accommodation demand symmetry and spatial frequency, were significant (see Results).

The influence of task difficulty (easy, difficult), SF (low, average and high: 1, 4 and 10 c/deg, respectively) and AD symmetry (i.e., whether the AD is the same for both eyes or not) on task accuracy (TA) is illustrated in Figure 3. As presented in the methods section, the task is denoted as “easy” when subjects had to compare gratings that had either the same orientation or a 30° difference, and “difficult” when they had to compare gratings that had either the same orientation or a 15° difference.

As expected, TA decreased when task difficulty increased (main effect: *F*(1,14) = 35.28, *p* < 0.001). The other main effects were significant, as well: AD symmetry *F*(1,14) = 25.71, *p* < 0.001, SF *F*(2,28) = 18.09, *p* < 0.001. The three-way interaction of difficulty by AD symmetry by SF was not significant (*F*(2,28) = 1.12, *p* = 0.34). Among the two-way interactions, only the one between AD symmetry and SF reached significance (*F*(2,28) = 5.19, *p* = 0.001; difficulty and SF: *F*(1,14) = 1.4, *p* = 0.26; AD symmetry and difficulty: *F*(1,14) = 4.46, *p* = 0.053).

### 3.2. Accommodation Response

**Figure 4:**
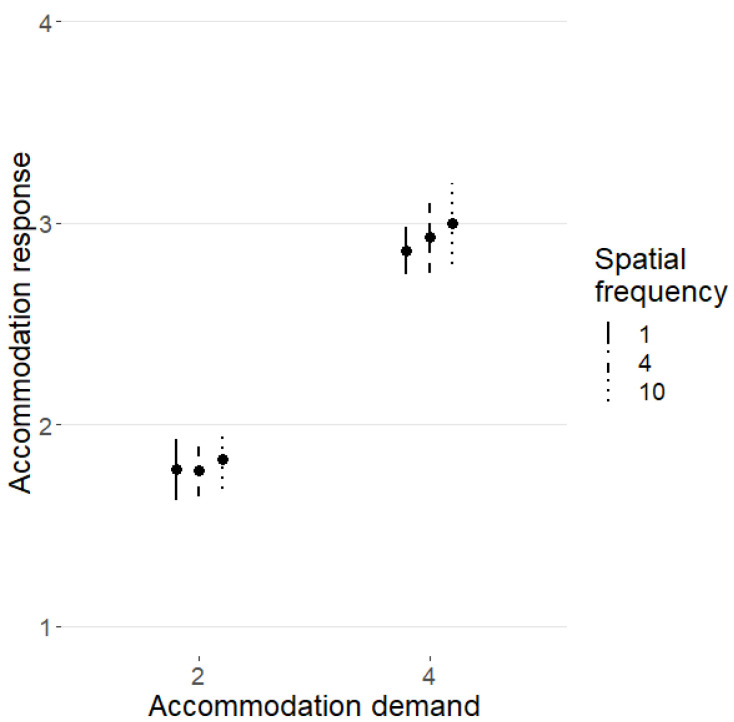
The effect of spatial frequency on accommodation response. Only data from isometropic conditions were used. The abscissa axis represents accommodation demand, and accommodation response is on the ordinate axis. The spatial frequency is denoted by the line type: solid line—1 c/deg; dashed line—4 c/deg; dotted line—10 c/deg.

We first consider the case when the AD for both eyes was the same. The influence of SF on AR, taken as the average response of the two eyes, is depicted in Figure 4.

AR was fitted to (only isometropic) AD, task difficulty and SF. Contrary to expectations, the only significant factor (including all interactions) was AD (*F*(1,14) = 118.7, *p* < 0.001). Then, we filtered out blocks with low accuracy (<75% correct responses); the data of three subjects were discarded because they did not reach 75% accuracy in any blocks in at least one condition. Again, AD was the only significant factor (*F*(1,11) = 72.36, *p* < 0.001). We found no significant effect of SF on AR, as the fine-focus control hypothesis would predict [15], with or without control filtering.

Because no statistically significant effect of SF or task difficulty on AR was found, the data across SF and task difficulty were pooled together for the following analysis.

**Figure 5:**
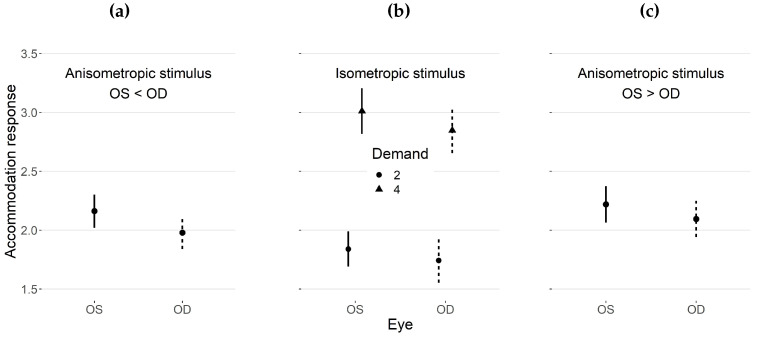
Accommodation response in the left (OS) and right (OD) eyes. The ordinate axis scales the accommodation response in diopters. A different line type is used for each eye: a solid line for the left eye and a dashed line for the right one. The three plots represent the four different conditions: (**a**,**c**) show the response to anisometropic stimuli; (**b**) shows the accommodative response to isometropic stimuli (2D with disks and 4D with triangles). The error bars represent 95% CI.

The impact of aniso-AD on the accommodation response of each eye is presented in Figure 5. Left and right eye ARs were close in all conditions, and there was no significant effect of AD difference on aniso-AR either before (*F*(2,28) = 0.51, *p* = 0.61) or after filtering (*F*(2,26) = 0.22, *p* = 0.81) (one subject was excluded from this analysis as they never reached 75% accuracy in any block in at least one of the conditions).

The differences between the anisometropic conditions (OS < OD and OS > OD) did not reach the level of significance (*F*(1,14) = 1.73, *p* = 0.21). However, the average responses of the two eyes differed between the isometropic and anisometropic conditions. The AR to isometropic 2D stimuli was significantly lower compared to anisometropic conditions when the AD was 4D in one eye (*F*(1,14) = 40.11, *p* < 0.001 for the right one, *F*(1,14) = 24.41, *p* < 0.001 for the left one).

## 4. Discussion

It was established a long time ago that the visual stimulus’ characteristics are not alone in determining AR [23]. Several umbrella terms seem to have been used for the “other” factors at play: proximal (following Maddox’s term for the same concept in vergence [24]) [25,26], spatiotopic (as opposed to retinotopic) [27], non-dioptric [28], psychological (as opposed to physiological) [29], non-optical [30], non-sensory and “top-down” [31]. These factors have been introduced into computational models of accommodation as singular operators [32,33]. There are a number of experiments investigating the possible non-sensory factors involved in accommodation, which include the anticipation of dynamic stimuli [34], previous knowledge about stimulus distance [32], voluntary control [14,35], instruction [15,31,36], mental effort [30], accommodative effort [15] and imagery [37].

In this study, we suggested that this “top-down” control effect could be the cause of some conflicting results concerning aniso-accommodation in the literature. We thus attempted to test if controlling for higher-level factors, in addition to usual stimulus properties (SF, AD) (and with a high enough number of subjects), would help clarify these contradictory results.

This was achieved (a) by requiring them to keep the images clear enough to perform the task, and (b) by filtering out blocks of trials with low TA, as they may coincide with insufficient AR.

The task consisted of comparing the orientations of two sinusoidal gratings presented dichoptically. Subjects initiated each block of tests once fusing on binocular stimuli was successful. Subjects’ accuracy in the task was in agreement with expectations, i.e., it decreased with difficulty (the smaller the difference in orientation between the two gratings, the more difficult the task) and with the amplitude of the difference in AD. It also decreased with higher SF and AD asymmetry.

This suggests that the task was not too easy (otherwise, accuracy would not vary) and that the reduced contrast produced by the different orientations or different viewing distances did not help the subjects judge the similarity between two gratings. Alternatively, the task could be judged to be too hard.

### 4.1. The Effect of Spatial Frequency, in Isometropic Stimuli, on Accommodation Response

In order to use the most appropriate stimulus for accommodation, we first tested the effect of SF on AR.

Conflicting results regarding the dependence of AR on SF have been reported in the literature. Some studies show support for the fine-focus control hypothesis (i.e., higher SF causes higher AR) [38], while other papers support the contrast control hypothesis (higher AR in average SF (3–5 c/deg)). Ciuffreda and Hokoda [15] explained this inconsistency through the effect of instruction. That is, outside cases of insufficient accommodative effort, the fine-focus control hypothesis explains measurements well.

Our results for isometropic stimuli show that AR depended, as expected, on the AD but did not vary with SF (with or without filtering), i.e., our subjects did not adjust their accommodation for stimuli with more detail. This could have been explained assuming a large enough depth of focus for the eyes [39], but this contradicts our finding that TA decreased with higher SF. Also, the fact that TA decreased with SF rules out the possible critique that 10 c/deg, chosen here for technical reasons (see Appendix B), was not high enough to stimulate accommodation.

Our results suggest that in some situations, sinusoidal gratings may not be a good stimulus for research on accommodation. Although they have been widely used to study accommodation [38,40,41,42,43], they can be an ambiguous accommodation stimulus when out of focus. In the case of sinusoidal gratings, accommodation adjustments do not allow the visual system to resolve grating stripes to sharp edges. This decreases the amount of information that can be obtained by changing the refraction power of the eye. In extreme cases, this may open the accommodation loop, in which case the optical qualities of the stimulus lose their driving force on accommodation, and the impact of voluntary control may take the lead [30]. This means that the effects of higher-level control over accommodation (instruction, effort and others), which we aimed to factor out using the visual task, may be particularly strong when viewing sinusoidal gratings.

A limitation of the current study was the same contrast used across all SF levels, which may have effectively reduced the perceived contrast in higher spatial frequency and, thus, affected the AR. However, in a recent article, Xu and colleagues [44] also did not show evidence for the effect of SF in children with contrasts adjusted for each SF according to contrast detection thresholds. It is noteworthy that this finding was in contradiction with an earlier paper by the same group [43], which showed decreasing AR with increasing SF in adults. However, lower SFs were underrepresented in the study, as the tested range started at as high as 2 c/deg.

### 4.2. Aniso-Accommodation

The aim of this study was to assess if anisometropic stimuli would elicit aniso-AR. Previous studies showed conflicting results, with only one demonstrating a difference in left and right eye AR [10]. We assumed that these conflicting results could be due to the differences in the experimental procedure, notably, in how it motivated subjects to apply accommodative effort. In our study, we attempted to control subjects’ accommodative effort by presenting them with a task and using it to ensure an appropriate AR.

According to our results, the AR in anisometropic stimuli was the same in the left and right eyes, with a value slightly higher than the smaller AD, and did not depend on whether the right or left stimulus was closer. Importantly, TA suffered in anisometropic conditions compared to the isometropic conditions. This indicates that the unadjusted accommodation was not sufficient for the task at hand, and the alternative explanation of a large enough depth of field allowing subjects to view both targets could be refuted.

Dichoptic stimuli in our procedure could lead to binocular rivalry [45]. In rivalrous conditions, the two percepts—the right eye’s and left eye’s gratings—occasionally switch brtween dominant and suppressed states. In our experimental procedure, one can see how, in the isometropic conditions, gratings of equal orientation would result in normal stereoscopic fusion, whereas unequal orientations would produce rivalry, signaling the correct response to the observer. In the anisometropic conditions, on the contrary, both equal and unequal orientations could lead to binocular rivalry, since one of the eyes, assuming consensual AR, always views a blurry image.

This raises the issue of the possibility of simultaneous viewing of two gratings. By extension, it calls into question the independent adjustment of the left and right eyes’ AR. In fact, Flitcroft and Morley [46] measured accommodation in subjects reporting a current dominant image and found that accommodation follows the dominant eye’s target AD. Even though they measured one eye’s accommodation while relying on AR consensuality, they showed convincingly how AR traces the dominant AD.

However, not all processing of the suppressed image is seized [47], which can be demonstrated, for instance, by a strong stimulus presented to the inhibited eye, breaking suppression and inverting dominance. Moreover, it has been proposed that stereoscopic and rivalrous processing co-occur and result from parallel processing pathways [48]. Therefore, while the dominant eye input is prioritized, it is not entirely inconceivable that the blurry input of the suppressed eye can, at the very least, contribute to adjusting accommodation.

In our experiment, participants did not report having issues with fusing two monocular targets. If they experienced binocular rivalry, they could attempt to compare the gratings sequentially by switching the dominant image. However, subjects were specifically instructed and trained not to do this. In addition, they may not have had very strong voluntary control over switching between two stationary monocular gratings [49]. Also, again, if this had been the case, then TA would have been similar in isometropic and anisometropic conditions (which was not the case). We believe that this presents evidence for at least partial fusion of targets by our subjects even in the anisometropic conditions.

In Marran and Schor’s study, subjects were given an unlimited training period dedicated to producing aniso-AR. It is unclear, however, what this training entailed. In view of the dominating literature contradicting their findings, one may presume that subjects learnt to elicit aniso-AR in a feed-forward fashion regardless of the presented stimuli (instead of improving feedback loop control over accommodation [50]).

The present study did not involve such prolonged training aimed at inducing aniso-accommodation because the goal was to investigate whether aniso-AR could be detected under “natural” conditions, that is, when the observer performs a task with a binocular anisometropic stimulus. This approach promotes a more ecologically valid view of the possibility of aniso-accommodation. It has the benefit of being directly applicable to typical scenarios involving anisometropic stimuli, such as, for instance, in head-mounted-display users. Training, however, deserves special attention, as it remains the most salient distinctive feature in Marran and Schor’s experiment. If the effect of training is to be addressed, then a particular form of training that forces subjects to combine left and right eye stimuli, such as dichoptic training [51], should be used.

A possible limitation of the present study was the narrow range and low variety of levels of independent variables (task difficulty, AD, anisometropic difference). The primary reason for this was to avoid eye strain in subjects. A potential solution could be to have shorter study sessions spread out over a few days with more breaks. Using a finer scale of AD asymmetry (values between 0D and 2D) could be of particular interest, as it would present more ecologically plausible anisometropic conditions. Increasing the range of SF is possible by using computer screens with more dots per mm. Alternatively, it would be possible to use minification optics so that the perceived pixel density observed through a lens would be higher but this could impact the measurement. Also, it would be interesting to see if using similar dichoptic procedures would yield different results with other types of stimuli. Square-wave gratings seem an obvious choice; however, they would require a very different experimental procedure, since at small target distances, oblique square gratings produce significant graphical artifacts. For instance, a Vernier acuity task with dichoptic stimuli would solve this issue.

## 5. Conclusions

Research involving accommodation measurements can benefit greatly from procedures which allow for tracking accommodation efficacy using subjects’ behavioral accuracy. Previous studies on accommodation modulation through stimulus detail or aniso-accommodation did not provide any metric of subjects’ effort to accommodate or the sufficiency of their accommodation. We believe that this is particularly important in studies dedicated to the oculomotor system, as there is still much to be learnt about how top-down effects are employed in its mechanisms. In the case of the present study, this procedure allowed us to draw clearer and more complete conclusions from the obtained data than would have been possible otherwise.

## Figures and Tables

**Figure A1:**
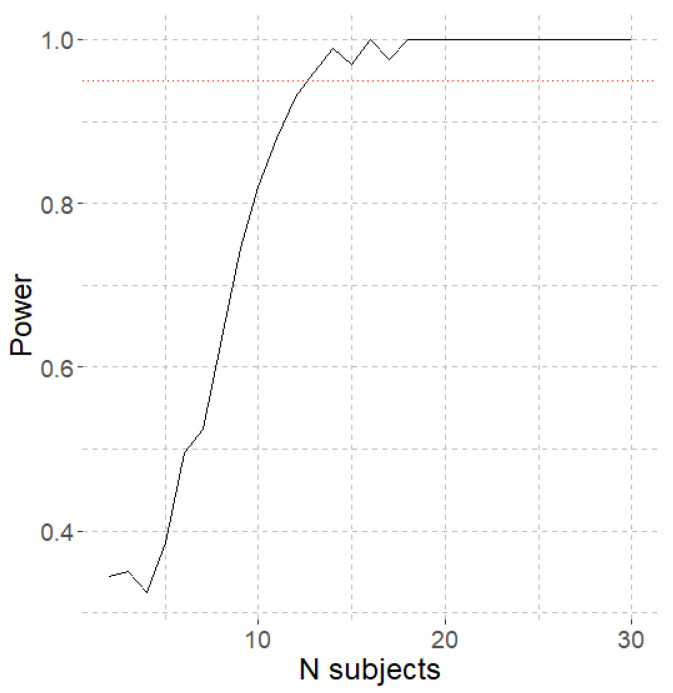
The results of the power analysis using the simulated data approach. The curve represents the average 1-*p* (ordinate axis) of 200 simulated experiments for a given sample size (abscissa axis). The red horizontal dotted line marks a power of 0.95.

## Data Availability

The data obtained in this experiment and the analysis script can be obtained from https://github.com/VasiliMarshev/Aniso-accommodation-study.git.

## Appendix A. Calculation of Required Sample Size for Aniso-Accommodation Hypothesis

We calculated the required sample size using a simulated data approach. To this end, we measured the baseline aniso-AR to isometropic stimuli in a pilot test, estimated the predicted aniso-AR in anisometropic conditions based on an existing study, produced simulated data based on these estimates and performed a statistical test analogous to what was used in the main experiment. By varying the number of simulated subjects, we calculated the probability of rejecting the null hypothesis, i.e., in there being no difference in the aniso-AR between isometropic and anisometropic conditions.

We recorded the AR of four subjects (two female) while they were performing the same task as in the main experiment in isometropic conditions (2D and 4D). Both gratings’ SF was either 4 c/deg or 10 c/deg. Aniso-AR in these conditions was used as the baseline below (M_iso_ = −0.01, *SD* = 0.46).

A series of experiments were generated with samples between 2 and 30 subjects. For isometropic conditions, six values were drawn from Gaussian distribution, with the average and SD obtained in the pilot study above for each simulated subject. For anisometropic conditions, we used the slope of 0.24 reported in [10] (M_L>R_ = 0.47, M_L<R_ = −0.49). Three conditions were produced in this manner: L = R; L > R; L < R.

For each sample size, 200 experiments were simulated. We calculated the average *p*-value of the test to produce the power with a given sample size (see Figure A1). The results of this power analysis suggested that 14 participants were required to reach a power of 0.95.

## Appendix B. The Choice of Spatial Frequencies

We chose in our experiment to use several SFs ranging between 1 c/deg and 10 c/deg. The highest SF in the present study was imposed by the small distance between the eyes and the screen and its pixel density. It was lower than what some previous studies used as high SFs: 15 c/deg [41], 16 c/deg [21,43], 19.2 c/deg [40], 30 c/deg [38]. However, since it is still considerably higher than the average band (3–5 c/deg) [40], we expected that it would be enough to elicit a response appropriate for a high SF.
